# Periosteal Osteosarcoma: A Review of 17 Cases with Mean Follow-up of 52 Months

**DOI:** 10.1080/1357714021000066368

**Published:** 2002-12

**Authors:** Matthew P. Revell, Neeta Deshmukh, Robert J. Grimer, Simon R. Carter, Roger M. Tillman

**Affiliations:** The Royal Orthopaedic Hospital Oncology Service Bristol Road South Northfield Birmingham B31 2AP UK

## Abstract

*Purpose:* Periosteal osteosarcomas are rare cartilage-rich bone tumours characterized by a juxtacortical eccentric position and are normally regarded oncologically as of intermediate to high grade.Their low incidence is mirrored by a small number of reported cases in the world literature. While there is general agreement that wide surgical excision is required, there is a paucity of evidence regarding adjuvant therapy. Previous reports have not indicated any consistent approach to this to allow appraisal.

*Patients and methods:* We report 17 cases treated at our centre over 16 years. Our policy was to use chemotherapy when the tumour showed any features of high grade.

*Results:* To date, no deaths have resulted from recurrence or metastasis of the tumour although there have been two deaths from other causes.

*Discussion:* Comparison of survival with existing studies is made to draw conclusions regarding future treatment of this condition in terms of surgical and adjuvant approaches.


*Sarcoma* (2002) **6**, 123–130

Taylor & Francis healthsciences

**REVIEW**

### Periosteal osteosarcoma: a review of 17 cases with mean follow-up of 52 months

MATTHEW P. REVELL, NEETA DESHMUKH, ROBERT J. GRIMER, SIMON R. CARTER & ROGER M. TILLMAN

*The Royal Orthopaedic Hospital Oncology Service, Bristol Road South, Northfield, Birmingham B31 2AP, UK*

**Abstract**

*Purpose:* Periosteal osteosarcomas are rare cartilage-rich bone tumours characterized by a juxtacortical eccentric position and are normally regarded oncologically as of intermediate to high grade. Their low incidence is mirrored by a small number of reported cases in the world literature. While there is general agreement that wide surgical excision is required, there is a paucity of evidence regarding adjuvant therapy. Previous reports have not indicated any consistent approach to this to allow appraisal.

*Patients and methods:* We report 17 cases treated at our centre over 16 years. Our policy was to use chemotherapy when the tumour showed any features of high grade.

*Results:* To date, no deaths have resulted from recurrence or metastasis of the tumour although there have been two deaths from other causes.

*Discussion:* Comparison of survival with existing studies is made to draw conclusions regarding future treatment of this condition in terms of surgical and adjuvant approaches.

### Introduction

Periosteal osteosarcoma is a well-defined pathological entity with characteristic radiological and histological features. First recognized by Ewing in 1939[1] it was only described as a separate entity in 1955 by Litchenstein.[2] Unni in 1976[3] produced the first large series about this condition, whilst Campanacci in the same year recommended wide excision to maximize cure.[4] It is a rare tumour, representing no more than 1–2% of all osteosarcomas and the number of patients appearing in the world literature is small.

Controversy remains about the appropriate treatment of periosteal osteosarcoma. All are agreed that complete surgical excision is mandatory for local control but the role of chemotherapy in this condition is still debatable. Previous analyses have reported relatively high numbers of tumour related deaths, particularly early studies.[3–7] The main identifiable risk factor for early death is inadequate margin of surgical excision. Only Hall *et al.* report no metastases in their sample of six patients.

We present a series of 17 patients treated at our centre over 16 years. Our patients received chemotherapy when the tumour showed high-grade histological features or when there was evidence of medullary involvement. To date no patients have died as a direct result of their osteosarcoma or of related treatment. We examine possible reasons for this encouraging survival rate and discuss the implications for therapeutic approaches in this condition.

### Patients and methods

In our unit, patient, diagnostic and treatment data are collected prospectively on a computer database. Review of this showed that 17 patients had been diagnosed as having a periosteal osteosarcoma between 1983 and 1999. There were 10 males and seven females with an age range from 10 to 35 years and a median of 18 years (Table 1). Five tumours arose in the proximal tibia and four in the proximal femur (Fig. 1).

All patients underwent a full staging procedure[8] with bone scintigraphy, CT scan of chest and MRI or CT of the tumour itself.

Radiological findings were consistent with a diagnosis of periosteal osteosarcoma in all cases, showing a tumour on the periosteal surface of the bone, with the epicentre outside the bone and typical stippled calcification (Fig. 2a,b).

Correspondence to: Mr R. Grimer, Consultant Orthopaedic Surgeon, The Royal Orthopaedic Hospital Oncology Service, Bristol Road South, Northfield, Birmingham B31 2AP, UK. Tel.: +44-121-685-4150; Fax: +44-121-685-4146; E-mail: rob.grimer@roh.nhs.uk

ISSN 1357–714X print/ISSN 1369–1643 online/02/0200123–08 © Taylor & Francis Ltd
DOI: 10.1080/1357714021000066368

**Table 1.** *Ages of patients with periosteal osteosarcoma*

| Decade of life | Number |
|---|---|
| 10–19 | 5 |
| 20–29 | 9 |
| 30–39 | 3 |
| 40–49 | 4 |

A histopathologist not originally involved in the diagnosis reviewed the specimens. Medullary involvement, bony and soft tissue resection margins, grade of tumour and percentage necrosis, if applicable, were confirmed. Features typical of periosteal osteosarcoma were considered to be: a tumour forming a lobulated pattern with malignant spindle cells at the periphery of the nodules. Centrally, the lobules had malignant cartilage containing areas of endochondral calcification and seams of osteoid (Fig. 3a,b).

All patients underwent surgery to excise the tumour. The primary surgical procedures used were simple excision in four cases, excision with bone graft in three or excision and insertion of an endoprosthesis in the remaining 10 (Table 2).

Fourteen of the 17 cases received chemotherapy, the usual regime being a combination of cisplatin and doxorubicin as would be used for conventional osteosarcoma.[9,10] Ten of the patients received neoadjuvant chemotherapy, whilst four only received it following surgical excision. Three patients did not receive chemotherapy where it was considered there would be no added benefit in addition to surgery. One patient received radiotherapy in addition to chemotherapy because of a high-grade tumour and close margins of excision.

Patient notes were analyzed with particular regard to operative management, other treatment modalities, recurrence, metastasis and death. Axial images were examined with a hand-held angle measure to quantify the circumferential extent of the tumour.

### Results

### *Biopsy results*

Of the 17 patients, seven had the diagnosis of periosteal osteosarcoma confirmed at the time of the original biopsy. Of these, four biopsies were done at our centre and three were done elsewhere. Ten patients had an initial inconclusive biopsy, the most common differential diagnosis being a periosteal chondroma. Confusion was sometimes apparent in patients having needle biopsies in which only small samples were obtained. In these cases, the diagnosis was only eventually confirmed following complete rebiopsy or excision of the lesion.

### *Tumour variables*

The maximum dimension of the tumour varied from 4 to 13.5 cm (mean 7.9 ± 2.7 cm). On axial imaging, the tumour was found to encircle between 5 and 100% of the cortex of the bone. Histologically all the tumours were intermediate or high grade.[11,12] Medullary involvement was demonstrable in four specimens. One contained demonstrable tumour in the profunda vein. Percentage necrosis was recorded for the tumours of those patients who had undergone chemotherapy preoperatively. Only two of these 10 patients who received neoadjuvent chemotherapy had greater than 90% necrosis.

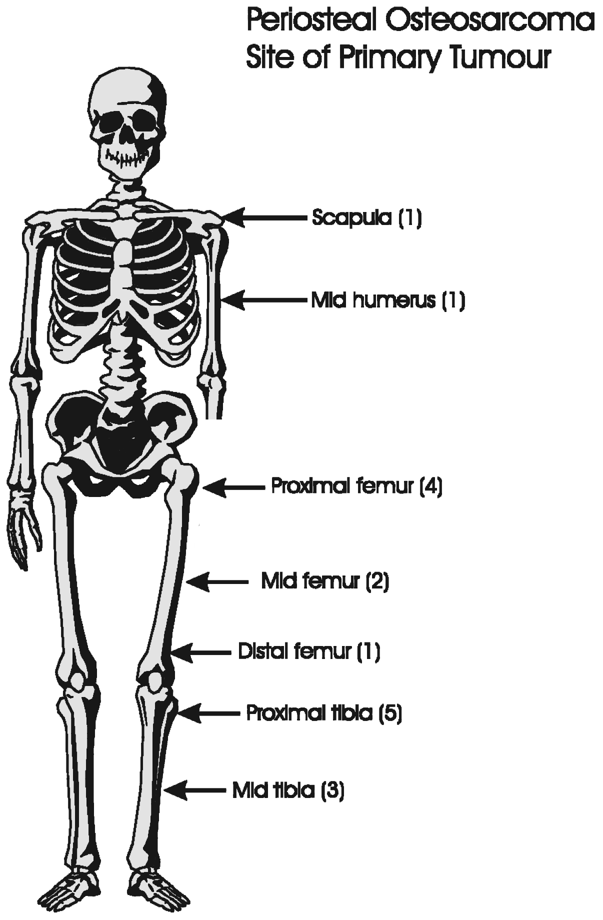

**Fig. 1.** *Site of tumour.*

### *Surgery*

Ten patients had complete excision of the tumour and replacement of the involved bone with an endoprosthesis as the first surgical intervention. The soft tissue margins of excision were often 1 mm or less as the muscles frequently 'fell away' from the surface of the tumour. The bony resection margins were judged clear in all cases.

Three patients underwent excision of the tumour without reconstruction in an attempt to 'shark bite' the tumour. In these cases, the bony margins of excision proved positive in two cases and, as a result, two have required re-excision and one has gone on to have a metal endoprosthesis. In those treated primarily with excision and bone grafting, the

(a)

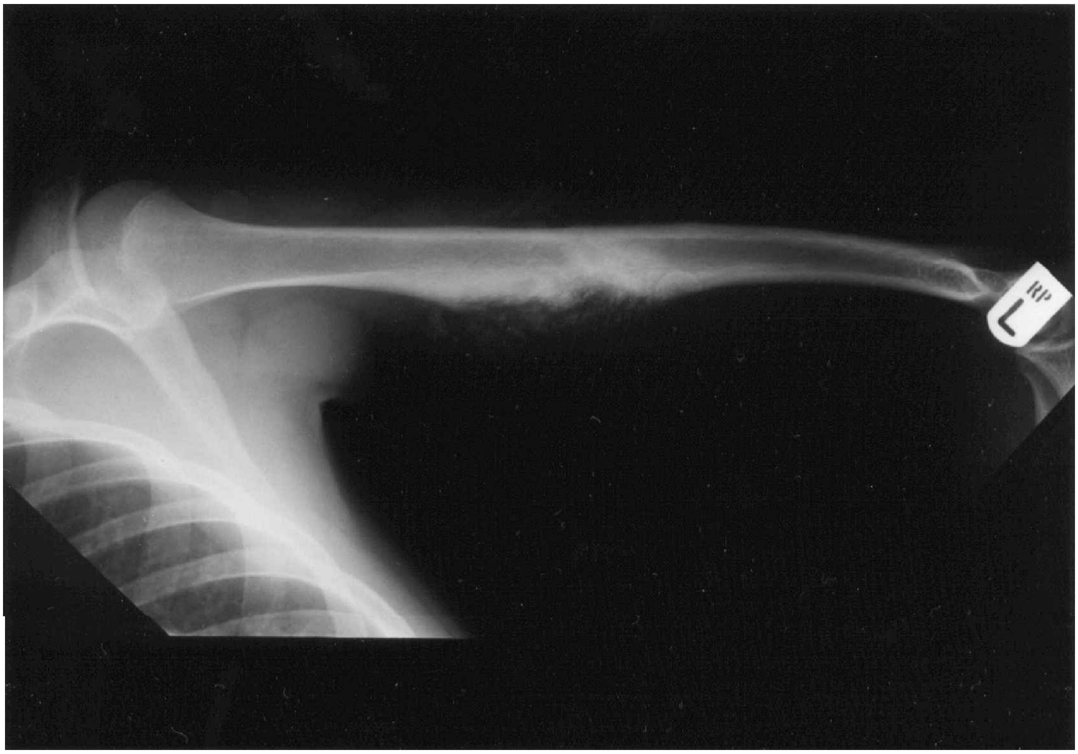

(b)

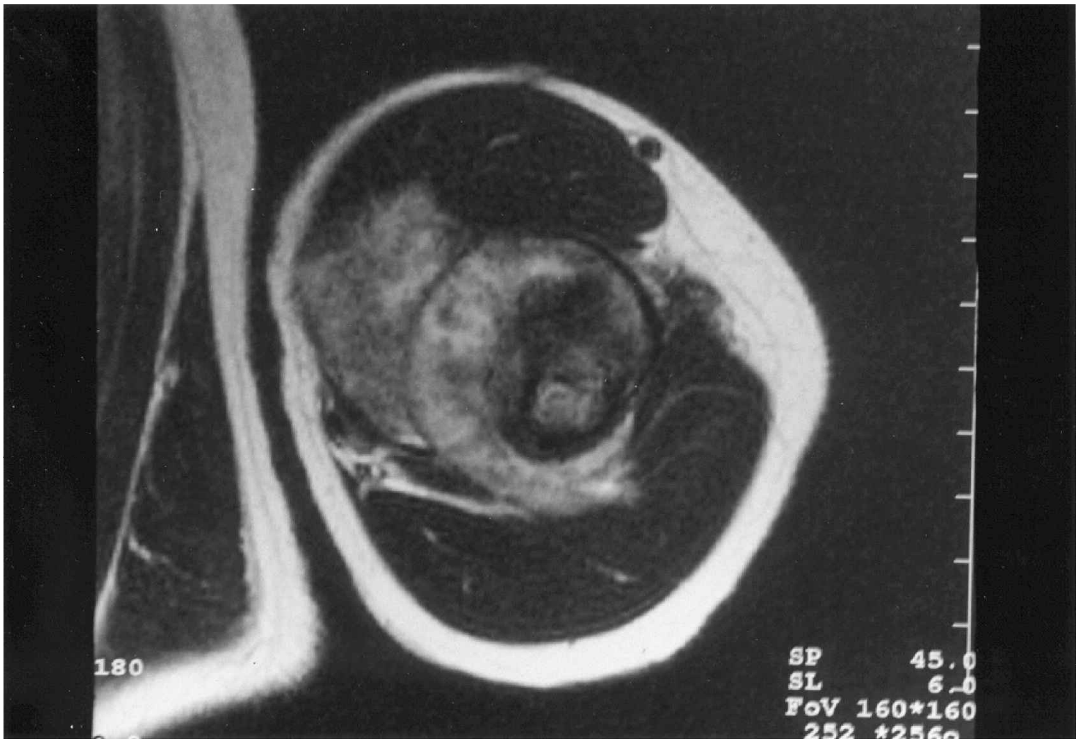

**Fig. 2.** *(a) Plain radiograph of a humerus showing many typical features of a periosteal osteosarcoma in the diaphyseal region. (b) Magnetic resonance image of the same tumour, showing eccentric epicentre and near encirclement.*

margins of excision were clear in all cases. One patient required internal fixation then endoprosthesis for non-union.

*Local control*

There was one case of local recurrence in a patient who had what was planned as a wide local excision but in whom the excision margins proved positive. He had chemotherapy postoperatively but subsequently developed local recurrence after 12 months at the site of the tumour excision margin. He then underwent a further complete resection of the involved bone with wide margins and insertion of an endoprosthesis. This became infected and he elected to have an above knee amputation.

**Table 2.** *Treatment and outcomes of 17 patients with periosteal osteosarcoma*

| No | Sex | Age | Site of tumour | Max length (cm) | Medullary | Chemo-therapy | % Necrosis | Soft tissue margin | Other involvement | Primary operation | Adverse events and deaths | Months alive |
|---|---|---|---|---|---|---|---|---|---|---|---|---|
| 1 | M | 15 | Proximal tibia | - | Yes | Yes | 90 | 2 mm | | Endoprosthesis | NED | 63 |
| 2 | M | 18 | Tuberosity tibia | 7 | Yes | None | - | Intralesional | | Excision | Wide re-excision + bone graft | 9 |
| 3 | F | 19 | Mid femur | - | No | None | - | 1 mm | | Endoprosthesis | NED | 18 |
| 4 | M | 13 | Proximal tibia | 7 | No | Yes | - | Wide | | Endoprosthesis | NED | 132 |
| 5 | M | 14 | Mid tibia | 6 | No | Yes | 40 | 15 mm | | Excision and bone graft | NED | 86 |
| 6 | F | 23 | Proximal tibia | 6 | No | Yes | 95 | Wide | | Endoprosthesis | NED | 195 |
| 7 | M | 10 | Scapula | 10 | No | Yes | - | <1 mm | | Excision | NED | 50 |
| 8 | M | 17 | Mid tibia | 8.5 | No | Yes | - | >5 mm | | Excision and bone graft | NED | 133 |
| 9 | F | 24 | Mid tibia | 7 | No | None | - | <1 mm | | Excision | NED | 25 |
| 10 | F | 26 | Proximal femur | 4 | No | Yes | 10 | 0.5 mm | | Endoprosthesis | NED | 99 |
| 11 | M | 22 | Proximal tibia | 5 | No | Yes | - | 1 mm | | Excision | Local recurrence 11 months, excision and endoprosthesis | 77 |
| 12 | M | 16 | Proximal femur | 10 | No | Yes | 60 | 1 mm | | Endoprosthesis | NED | 12 |
| 13 | F | 23 | Proximal femur | 5 | No | Yes | 80 | 1 mm | | Endoprosthesis | NED | 93 |
| 14 | F | 35 | Distal femur | 13 | Yes | Yes | 30 | <1 mm | | Endoprosthesis | Died brain tumour 10 years post operation | 121 |
| 15 | M | 15 | Mid femur | - | No | Yes | 70 | 1 mm | Profunda vein | Endoprosthesis | NED | 50 |
| 16 | F | 18 | Proximal femur | 12.5 | No | Yes | 70 | >5 mm | | Endoprosthesis | Died AML 10 years | 128 |
| 17 | M | 15 | Mid humerus | 9.5 | Yes | Yes | 20 | 1 mm | | Excision and bone graft | NED | 47 |

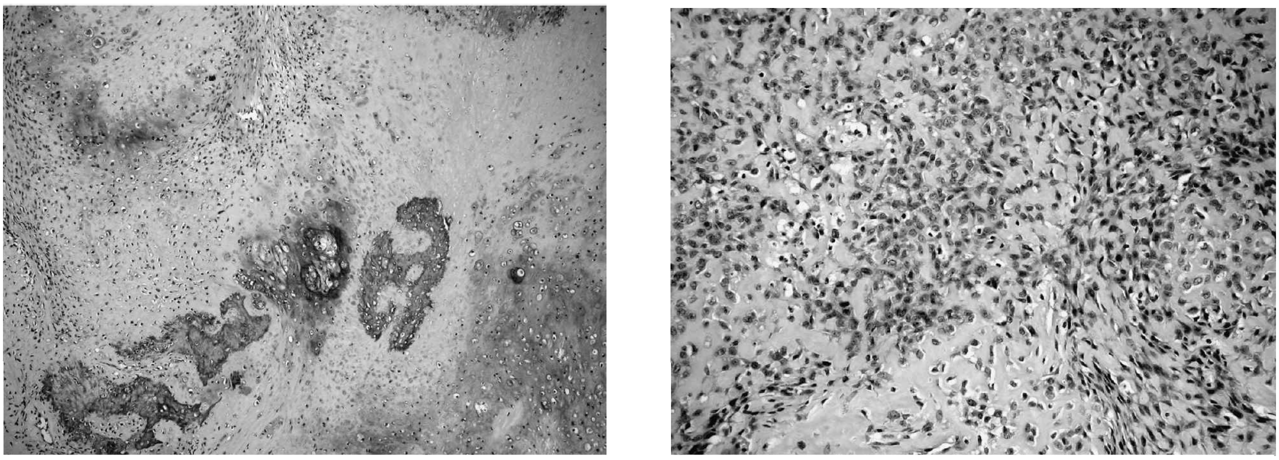

**Fig. 3.** *(a) Lobules of neoplastic cartilage and primitive bone matrix undergoing mineralisation. The tumour is of intermediate grade (H&E stain, (40)). (b) High-grade tumours also showed undifferentiated mesenchymal cells with osteoid matrix deposition (H&E stain, (100)).*

*Survival*

The mean follow-up is 82 ± 53 months (range 9–200 months). No patient has developed metastases to date. Fifteen of the 17 patients are still living. One patient developed acute myeloid leukaemia 10 years following chemotherapy and endoprosthetic replacement of the proximal femur. A second patient, who had also undergone proximal femoral endoprosthesis and chemotherapy, died 10 years later of a primary brain tumour.

### Discussion

The definition of periosteal osteosarcoma is very precise.[13] Importantly it is essential to exclude high-grade surface osteosarcomas and parosteal osteosarcomas from this group. We did not exclude patients with medullary involvement from the study. In cases where the histological and radiological findings were otherwise typical they were included for analysis. Unni *et al.* originally included no cases where medullary involvement was present. Hall *et al.*, however, argued persuasively that medullary involvement did not preclude a diagnosis of periosteal osteosarcoma. If the possibility of including cases of intramedullary osteosarcoma in the group is entertained, this could reasonably be expected to elevate the average grade of tumour and adversely affect the survival statistics.

Our survival figures are very encouraging and our 100% metastasis-free survival during a mean follow-up over 10 years represents one of the best survival figures yet published. The reasons for this are not immediately apparent but may well be due to two factors. Firstly the radical resection of the tumour, preventing local recurrence which is known to be a risk factor for the subsequent development of metastatic disease in high-grade osteosarcoma[14] and secondly the use of chemotherapy.

Many authors have highlighted the high incidence of local recurrence in periosteal tumours when complete excision is not achieved. The natural tendency to try and preserve the patients bone by doing a ‘shark-bite’ procedure may risk leaving tumour behind, as was found in two of our cases where this was attempted. One of the problems with this sort of surgery is actually assessing the true extent of the tumour in the subperiosteal plane and even with axial MRI and CT this can be difficult. It is likely that the tumour spreads further than can be imaged using these modalities and hence wider margins than might at first be considered are mandatory for safety. Masterson *et al.* described this procedure as the ‘geometric osteotomy’ but only had one periosteal osteosarcoma in their series of 12 cases.[15] They achieved negative margins in all their cases but had one local recurrence in a periosteal chondroma. The average percentage of cortex circumference involved in their series was 30%, although one patient had 50% involvement. In our group, the percentage circumference varied more greatly from a small cortical lesion to complete encirclement.

Our low rate of local recurrence is, however, at the expense of a large proportion of patients undergoing limb salvage surgery with endoprostheses. These provide a good guarantee of local control and early return of function, but do increase the long-term risks of problems with the limb due to complications such as infection, loosening and prosthetic failure.[16–19] The relative risks of cure of the disease need to be balanced with the long-term risks to the limb with these methods of limb salvage. Given the uncertainty of outcome with local resection and the improving results of major resection and limb salvage surgery, we believe that every attempt to obtain wide margins at the first surgical procedure should be made.

Our patient group shows improved survival when compared with previously reported series. Rasquinha *et al.* reported on a very similar series to ours and had three local recurrences and three deaths, of whom two were due to other causes.[20] They too suggested

**Table 3.** *Summary of previous publications with regard to adjuvant therapy and survival data (figures derived from original publications by the present authors unless otherwise stated)*

| Author and year | Cases | Complete excision | Primary systemic chemotherapy | Other adjuvant therapy | Local recurrence | Metastases | Time of follow-up | Overall survival | Tumour-related deaths |
|---|---|---|---|---|---|---|---|---|---|
| Unni *et al.*,1 1976 | 23 | 78% (18) | 0% (0) | Radiotherapy to lungs | 30% (7) | 17% (4) | 11 months –29 years | 65% | 14% (5) |
| Campanacci and Giunti[2], 1976 | 28 | Not recorded | Not recorded | | ? 40% (15/41) | 36% (10) | 3-56 years | 55%3 | 36% (10) |
| Bertolini *et al.*,[7] 1982 | 20 | 65% (13) | 10% (2) | Radiotherapy | 35% (7) | 15% (3) | 9 months –39 years | 85% | 15% (3) |
| Hall *et al.*,[6] 1985 | 6 | 84% (5) | 17% (1) | Femoral artery adriamycin, radiotherapy | 17% (1) | 0% (0) | 16–60 years | 84% | 16% (1) |
| Ritts *et al.*,[5] 1987 | 22 | 91% (20) | 9% (2) | Radiotherapy | 14% (3) | 14% (3) | 1.8–29.3 years | 68% | 18% (4) |
| Rasquinha *et al.*,[20] 1999 | 16 | 81% (13) | Not stated | | 19% (3) | Not stated | Up to 215 months | 81% | 6% (1) |
| This paper | 17 | 94% (16) | 82% (14) | Radiotherapy | 6% (1) | 0% (0) | 1–16 years | 88% | 0% (0) |

1 Unni is a co-author of Ritts and both report series from the Mayo Clinic
2 Campannacci is a co-author of Bertolini and both report from Bologna
3 Author's quoted survival. It is not possible to corroborate this from the figures given

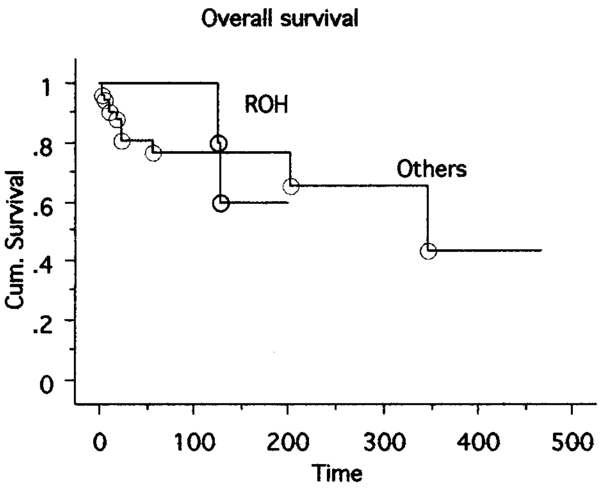

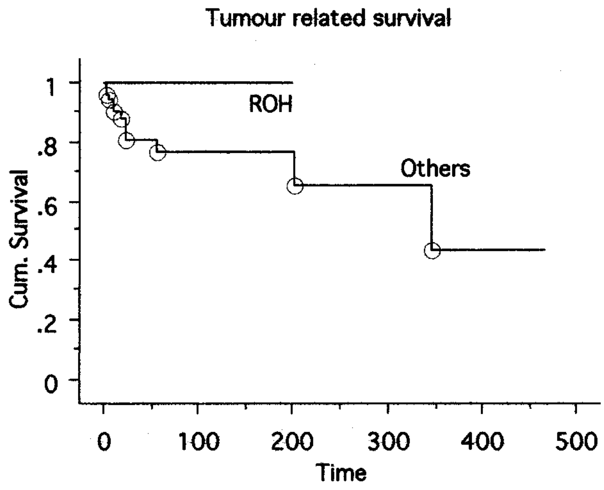

**Fig. 4.** *Survival from periosteal osteosarcoma. (a) Overall survival for 66 patients with published data. No deaths after 120 months were due to the primary tumour. (b) Tumour-free survival from diagnosis comparing this series with data from all previous published series.*

that chemotherapy was valuable but emphasized the importance of complete surgical excision. Figures of local recurrence and survival, as far as they are comparable, are summarized in Table 3. Survival curves derived from original data are shown in Fig. 4.

One noticeable feature in many series, including our own, is the high incidence of deaths from causes other than osteosarcoma. A death from acute myelocytic leukemia (AML) is reported in our own series and there are two cases of AML in the account of Ritts *et al.* One of our patients died of a brain tumour, [21] although it has not been possible to elucidate the histological type. It seems likely that environmental or genetic predisposition to tumours accounts for the majority of these cases. It is of particular interest that one of our patients came from a 'p53 family', a sibling having previously been treated for a brain tumour. Another possibility is that treatments have given rise to an increased risk of malignancy in later life. However, the inconsistency between centres of adjuvant therapies does not lead the authors to the conclusion that such a pattern can be found from the data so far.

We believe that the impressive disease-free survival figures in this series are a reflection of the aggressive surgical resection policy combined with the use of chemotherapy for all patients with high-grade elements of the tumour. Historical series have suggested that overall survival is about 70% without chemotherapy, unfortunately it has thus far proved impossible to identify the 30% of patients who are the ones most at risk and who thus might benefit from chemotherapy. Until this is clear-cut, it would seem prudent to continue to treat all patients with high-grade areas or medullary involvement with this combination of chemotherapy and wide surgical resection. It is unlikely that a randomized study will ever answer this question for such a rare disease. Similarly, the role of neo as opposed to adjuvant chemotherapy is unclear but, given the tendency to treat all high-grade osteosarcoma with neoadjuvant chemotherapy, it seems sensible to do the same for all periosteal osteosarcoma with a biopsy-proven diagnosis.

### References

1 Ewing J. A review of the classification of bone tumours. *Bull Am Coll Surg* 1939; 24: 290–5.

2 Lichtenstein L. Tumours of periosteal origin. Cancer 1955; 8: 1060–9.

3 Unni KK, Dahlin DC, Veabout JW. Periosteal osteogenic sarcoma. *Cancer* 1976; 37: 2476–85.

4 Campanacci M, Giunti A. Periosteal osteosarcoma. It J Orthop Trauma 1976; 2: 23–35.

5 Ritts GD, Pritchard DJ, Unni KK, Beabout JW, Eckhardt JJ. Periosteal osteosarcoma. *Clin Orthop Relat Res* 1987; 219: 299–308.

6 Hall B, Robinson L, Malawar M, Dunham WK. Periosteal Osteosarcoma. *Cancer* 1985; 55: 165–71.

7 Bertolini F, Boriani S, Laus M, Campanicci M. Periosteal Chondrosarcoma and Periosteal Osteosarcoma. *J Bone Jt Surg* 1982; 64B(3): 370–6.

8 Enneking WF. A system of staging musculoskeletal neoplasms. *Clin Orthop* 1986; 204: 9–24.

9 Bramwell VHC, Burgers M, Sneath RSEA. A comparison of two short intensive adjuvant chemotherapy regimens in operable osteosarcoma of limbs in children and young adults: The first study of the European Osteosarcoma Intergroup. *J Clin Oncol* 1992; 10: 1579–91.

10 Souhami RL, Craft AW, Van der Eijken JW, Nooij M, Spooner D, Bramwell VHC, Wierzbicki R, Malcolm AJ, Kirkpatrick AL, Uscinska BM, van Glabbeke M, Machin D. Randomised trial of two regimes of chemo-therapy in operable osteosarcoma: a study of the European Osteosarcoma Intergroup. *Lancet* 1997; 350: 911–7.

11 Unni KK, Dahlin DC. Grading of bone tumours. Semin Diag Pathol 1984; 1: 165–72.

12 Meister P, Konrad E, Lob G, Janka G, Keyl W, Sturz H. Osteosarcoma. Histological evaluation and grading. *Arch Orthop Trauma Surg* 1979; 94: 91–8.

13 Mirra JM, Picci P, Gold RH. *Bone tumours: clinical, radiologic and pathologic correlations.* Philadelphia, PA: Lea and Febiger, 1989.

14 Weeden S, Grimer RJ, Cannon SR, Taminiau AH, Uscinska BM, I European Osteosarcoma. The effect of local recurrence on survival in resected osteosarcoma. *Eur J Cancer* 2001; 37(1): 39–46.

15 Masterson EL, Ferracini R, Davia AM, Wunder JS, Bell RS. The geometric osteotomy: joint preservation in juxta-articular surface bone neoplasms. *Sarcoma* 1997; 1: 167–74.
16 Roberts DF, Chan D, Grimer RJ, Sneath RS, Scales JT. Prosthetic replacement of the distal femur for primary bone tumours. *J Bone Jt Surg* 1991; 73B: 762–9.
17 Kabukcuoglu Y, Grimer RJ, Tillman RM, Carter SR. Endoprosthetic replacement for primary malignant tumors of the proximal femur. *Clin Orthop Relat Res* 1999; 358: 8–14.
18 Grimer RJ, Carter SR, Tillman RM, Sneath RS, Walker PS, Unwin PS, Shewell PC. Endoprosthetic replacement of the proximal tibia. *J Bone Jt Surg* 1999; 81B(3): 488–94.
19 Unwin PS, Cannon SR, Grimer RJ, Kemp HBS, Sneath RS, Walker PS. Aseptic loosening in cemented custom made prosthetic replacements for bone tumours of the lower limb. *J Bone Jt Surg* 1996; 78B: 5–13.
20 Rasquinha VJ, Zambakidis C, Pringle JAS, Saifuddin A, Briggs TWR, Cannon SR. Periosteal osteosarcoma. *J Bone Jt Surg* 1999; 81B (Suppl II): 186.
21 Kleihues P, Schauble B, zur Hausen A, Esteve J, Ohgaki H. Tumours associated with p53 germline mutations, a synopsis of 91 families. *Am J Pathol* 1997; 150(1): 1–13.

## References

[PDF_00381] Bertoni F., Boriani S., Laus M., Campanacci M. (1982). Periosteal chondrosarcoma and periosteal osteosarcoma. Two distinct entities.. J Bone Joint Surg Br.

[PDF_00386] Bramwell V. H., Burgers M., Sneath R., Souhami R., van Oosterom A. T., Voûte P. A., Rouesse J., Spooner D., Craft A. W., Somers R. (1992). A comparison of two short intensive adjuvant chemotherapy regimens in operable osteosarcoma of limbs in children and young adults: the first study of the European Osteosarcoma Intergroup.. J Clin Oncol.

[PDF_00374] Campanacci M., Giunti A. (1976). Periosteal osteosarcoma. Review of 41 cases, 22 with long-term follow-up.. Ital J Orthop Traumatol.

[PDF_00384] Enneking W. F. (1986). A system of staging musculoskeletal neoplasms.. Clin Orthop Relat Res.

[PDF_00429] Grimer R. J., Carter S. R., Tillman R. M., Sneath R. S., Walker P. S., Unwin P. S., Shewell P. C. (1999). Endoprosthetic replacement of the proximal tibia.. J Bone Joint Surg Br.

[PDF_00379] Hall R. B., Robinson L. H., Malawar M. M., Dunham W. K. (1985). Periosteal osteosarcoma.. Cancer.

[PDF_00425] Kabukcuoglu Y., Grimer R. J., Tillman R. M., Carter S. R. (1999). Endoprosthetic replacement for primary malignant tumors of the proximal femur.. Clin Orthop Relat Res.

[PDF_00441] Kleihues P., Schäuble B., zur Hausen A., Estève J., Ohgaki H. (1997). Tumors associated with p53 germline mutations: a synopsis of 91 families.. Am J Pathol.

[PDF_00370] LICHTENSTEIN L. (1955). Tumors of periosteal origin.. Cancer.

[PDF_00417] Masterson E. L., Ferracini R., Davis A. M., Wunder J. S., Bell R. S. (1997). The geometric osteotomy: joint preservation in juxta-articular surface bone neoplasms.. Sarcoma.

[PDF_00400] Meister P., Konrad E., Lob G., Janka G., Keyl W., Stürz H. (1979). Osteosarcoma: histological evaluation and grading.. Arch Orthop Trauma Surg.

[PDF_00376] Ritts G. D., Pritchard D. J., Unni K. K., Beabout J. W., Eckardt J. J. (1987). Periosteal osteosarcoma.. Clin Orthop Relat Res.

[PDF_00421] Roberts P., Chan D., Grimer R. J., Sneath R. S., Scales J. T. (1991). Prosthetic replacement of the distal femur for primary bone tumours.. J Bone Joint Surg Br.

[PDF_00392] Souhami R. L., Craft A. W., Van der Eijken J. W., Nooij M., Spooner D., Bramwell V. H., Wierzbicki R., Malcolm A. J., Kirkpatrick A., Uscinska B. M. (1997). Randomised trial of two regimens of chemotherapy in operable osteosarcoma: a study of the European Osteosarcoma Intergroup.. Lancet.

[PDF_00372] Unni K. K., Dahlin D. C., Beabout J. W. (1976). Periosteal osteogenic sarcoma.. Cancer.

[PDF_00398] Unni K. K., Dahlin D. C. (1984). Grading of bone tumors.. Semin Diagn Pathol.

[PDF_00433] Unwin P. S., Cannon S. R., Grimer R. J., Kemp H. B., Sneath R. S., Walker P. S. (1996). Aseptic loosening in cemented custom-made prosthetic replacements for bone tumours of the lower limb.. J Bone Joint Surg Br.

[PDF_00406] Weeden S., Grimer R. J., Cannon S. R., Taminiau A. H., Uscinska B. M., European Osteosarcoma Intergroup (2001). The effect of local recurrence on survival in resected osteosarcoma.. Eur J Cancer.

